# Introduction

**DOI:** 10.4103/0973-1229.32147

**Published:** 2007

## Academia-Industry Connect: Value, But With Significant Costs

### Value

As private investment in biomedical research has increased over the last few decades, both industry capital (venture or otherwise) and academia have been quick on the uptake. Huge inflows from the former, and technology transfer offices set up by the latter, have resulted in major academia-industry tie-ups. This is not an isolated phenomenon, for such tie-ups have sprung up in many other fields too. At most places they have been welcomed as the next best thing to technology itself.

There are many benefits of this connect. Much of the intellectual talent from academic institutions is getting absorbed in lucrative positions in industry. This is important, as the academic job scenario remains less than optimal. Industry is contributing massive research funds along with huge amounts as taxes, both of which ease pressure on the exchequer. Academia now has clear-cut employment options before it. Students and faculty can look forward to rewarding careers in industry. A general climate of optimism prevails all around. Applied research finds willing collaborators in venture capital funded industry, so a symbiotic growth is ensured for both. Stossel (2005) ventilates this position eloquently:

Biomedical research takes place in university health centers, in government laboratories and in the laboratories of pharmaceutical and medical-device companies, but only industry translates the research into products. Until the 1970s, academic researchers rarely worked on applied technologies, although they conducted clinical trials for companies and industry exploited academic basic research. Then, the revolution in molecular genetics that enabled investigators to produce large quantities of rare molecules with medicinal properties brought these groups closer together. Academic researchers joined venture capitalists in founding the biotechnology industry, leading to immense benefits - for example, the hepatitis B vaccine. The participation of prominent scientists in the first biotechnology companies instantly reversed the perception that academicians′ involvement in business activities was unsavory or evidence of intellectual bankruptcy. Nor were financially bankrupt university researchers receiving research support and profiting personally from their discoveries, although the value to society of the products far exceeded any individual's accrual of wealth. Universities also profited and created offices for filing patents and licensing intellectual property to companies. Congressional edicts encouraged these activities, the number of patents awarded to academic institutions increased enormously and promising products discovered by academicians continually enter development by industry. By any measure, the interactions between academic research and industrial research and development, as epitomized by biotechnology, have been overwhelmingly positive. We should celebrate their achievements and protect the process that led to them (Stossel, 2005).

### Significant Costs

While looking at the plus points, we cannot forget there are significant costs involved too. Major areas of conflict of interest have arisen, especially applicable to biomedical research as academia interacts with industry, which we have detailed in earlier monographs (Singh and Singh, 2005; Singh and Singh, 2005-2006). Similarly, there are some alarming fallouts that promise to dull the enthusiasm of even the most optimistic proponent of the academia-industry connect. They are related to disputes over patents and royalty, hostile encounters between academia and industry, as also between public and private enterprise, legal tangles, research misconduct of various types, antagonistic press and patient-advocate lobbies and a general atmosphere in which commercial interest get precedence over patient welfare.

Moreover, the caution of careful watchers of the Academia-Industry connect need to be considered here too. Gelijns and Their (2002), while acknowledging that, ‘*University-industry research collaborations have been key to a continued high degree of technological innovation in medicine*,’ also caution that, ‘*universities and industry need to maximize the upsides of collaboration and minimize the downsides by means of internal organizational change*’ and very insightfully mention, ‘*The costs of the university-industry interface might exceed the benefits, if the cultural and ethical principles of one partner overwhelm those of the other*.’ Similarly, Johns *et al* (2003), while similarly accepting the great benefit of academia-industry collaboration caution about the erosion of public trust in biomedical research: *‘Economic partnerships between industry and academia accelerate medical innovation and enhance patient access to medical advances, but such partnerships have sometimes eroded public trust in the research enterprise.*’ Their recommendation is to encourage only as much financial incentives as are beneficial to scientific advancement and none which are not: *‘Our recommendations are designed to address institutional conflicts of interest in a real and meaningful way, without damaging the incentive structures that have fostered so many scientific advances. Financial and nonfinancial incentives spur innovation. Industry-academia relationships are permitted only when there is a legitimate justification for them. In particular, no legitimate justification exists when a relationship with industry serves only the pecuniary interests of the holder, without directly and materially furthering scientific advancement. When industry-academia relationships promise to advance science, management of any potential conflicts of interest via independent and expert committees is necessary*.’

## The Purpose Of This Academia-Industry Symposium

This academia-industry symposium is an attempt to look at some of the issues that are likely to further dull our enthusiasm about the academia-industry connect if proper remedial measures are not undertaken rather urgently. It is important that rosy pictures being painted all around do not carry us away. Even if the painting is by the most well meaning. At the same time we must not paint gray that which is actually in colour. However, certain tones with such potential must be clearly discerned, before they blacken the whole picture. Even if they are disconcerting to watch, for preventive steps today may avoid costly errors later.

It is also necessary a full and proper cost calculation be carried out so an informed decision can be taken on how far the academia-industry connect can go and where it crosses legitimate boundaries. This Academia-Industry series has been an attempt to do some of this much-needed cost calculation.

## Earlier Monographs

In the earlier monographs, we had seen how academia-industry relationships have been strengthened, what have turned out to be the major areas of conflict during this strengthening and how less than desirable industry influence have caused concern to conscientious institutional players (as also, probably, to long term players from industry). An earlier monograph discussed issues like evidence of doctoring of research findings, selective publishing and delayed publication, underreporting, problems of incomplete disclosure of conflicts of interest, difficulties in multi-centered trials, ghost writing and duplicate publication, access to data and control over publication, negative drug trials and the porcupine dance between academia and industry, law suits against industry etc (Singh and Singh, 2005). We ended it with discussion on design and control of publication and the connection between funding and positive findings. We noted that an area of pressing concern was when industry decided not only to suppress unfavourable findings, but to both design and control publication of research in general (Singh and Singh, 2005) and of clinical trials in particular, because it made commercial sense for large drug companies to create their own study designs (Baird, 2003).

Also related was a strong connection between funding and positive findings for the sponsoring company's product (Singh and Singh, 2005). A number of studies and literature reviews (Davidson, 1986; Deyo *et al*, 1997; Stelfox *et al,* 1998; Rennie, 1999; Friedberg *et al*, 1999; Bekelman, Li and Gross, 2003) showed that industry funding was very systematic and there was a strong correlation between funding by manufacturers and findings that showed results supportive in terms of efficacy and safely of the sponsor's product (Baird, 2003).

## Voluntary Moratorium And Law-suits

In the monograph that followed (Singh and Singh, 2005-2006), we proposed a voluntary moratorium of industry spending over pampering of docs, as the wants of the latter were ever increasing and industry may feel satisfying them was an easy way to get their way through. However, increasing product costs to offset such ever-rising expense met with stiffer resistance from end users: the patients and insurance payers. Moreover, lawsuits against industry because of various acts of omission and commission of industry were on the upswing and had ominous portents:

GlaxoSmithKline agreed to pay US $ 2.5 million for charges that it suppressed findings that showed its antidepressant, Paxil, was harmful in children. It paid $75 million for allegedly overcharging patients and insurers for its anti-inflammatory drug, Relafen. Again agreed to pay US $ 92 million to end law suits over Augmentin, its antibiotic. Bayer settled over 2000 cases brought up against its drug Baycol, at a cost of US $ 800 million. Pfizer paid US $ 430 million to settle claims against off-label use of its drug, Neurontin. It is being sued at present by consumer groups claiming that the company misleadingly marketed its blockbuster cholesterol-lowering drug, Lipitor (Datamonitor Newswire, 30 Sept 2005; See also Wilke and Henley, 2005). Bristol-Myers Squibb promised to pay US $ 300 million to fend off a lawsuit by its own shareholders. In 2003, AstraZeneca settled criminal fraud charges of US$ 355 million in a case dealing with its drug Zoladex (Peterson, 2003). On July 14, 2004, Schering-Plough pleaded guilty of and was fined US $ 350 million in part for providing ‘educational grants’ to physicians, which were more appropriately called ‘kickbacks’ by the prosecutors (Harris, 16 July, 2004b). It faces an ongoing investigation whether it used sham consulting arrangements and clinical trials to remunerate doctors for writing its hepatitis drug, Intron A (Harris, 27 June, 2004a). TAP pharmaceuticals, a joint venture of Takeda Chemical Industries and Abbott Laboratories, entered into a settlement and paid US $ 290 million in criminal fines, plus US $ 585 million in civil penalties, out of which US $ 100 million went to whistle blowers (United States v. TAP Pharmaceuticals, Dec. 14, 2001). It continues to face further lawsuits by insurers and patients for unnecessary and costly services. All related to the way it used urologists to promote its Lupron, a potent gonadotropin-releasing hormone used in treating prostrate cancer. Wyeth has had a US $ 1 billion verdict against it for its Pondimin, an anti-obesity drug. The most recent Vioxx catastrophe is likely to result in a US $ 10-15 billion litigation bill (Horton, 2004) for the company involved, Merck, and probably cripple both its financial status as well as its reputation beyond repair (Singh and Singh, 2005-2006; Wilke and Henley reference added).

We had also predicted that greater squeeze on profit margins were to be expected, which would result in cost escalation with resultant resistance by patients and their advocates and a consequent downward spiral, both in economic viability and professional respectability, for industry and related academia. Hence we had proposed a voluntary moratorium on spending by industry. More of that later.

## Three Models For Future Growth Of Medicine

At that time, we had also proposed looking at three models for the future growth of medicine. First, whether it could become a corporate enterprise, regulated by the rules of business; second, whether it should remain a patient welfare centered profession, regulated by the rules of a profession; and third, whether a meeting ground between the two could be found and medicine could become a patient welfare centered professional enterprise, regulated by the rules of a profession where business considerations could not be sidelined, but accepted within an overarch of biomedical advance and patient welfare (Singh and Singh, 2005-2006).

In this monograph, we propose to carry on from the 2005 work mentioned above (Singh and Singh, 2005), to consider the manner in which clinical practice guidelines and Foundations are being influenced, how negative findings are suppressed and drugs hyped in the light of recent findings about drugs like Trasylol and Xigris, what concerns journal editors and associations of academia voice, what is the image of pharma and what can be done to brighten it and how industry has probably influenced the paradigm shift towards biological psychiatry.

Understanding the dynamics of such processes may hopefully help us decide between the three models of medicine mentioned in the earlier paragraph here and discussed in some detail in the later monograph (Singh and Singh, 2005-2006).
